# Immune Status of Workers with Professional Risk of Being Affected by Chrysotile Asbestos in Kazakhstan

**DOI:** 10.3390/ijerph192114603

**Published:** 2022-11-07

**Authors:** Sholpan Koigeldinova, Alexey Alexeyev, Zhengisbek Zharylkassyn, Yertay Otarov, Bauyrzhan Omarkulov, Magzhan Tilemissov, Chingiz Ismailov

**Affiliations:** 1 Department of Internal Diseases, Karaganda Medical University, 40 Gogol street, Karaganda 100008, Kazakhstan; 2 Institute of Public Health and Professional Health, Karaganda Medical University, 15 Mustafin street, Karaganda 100008, Kazakhstan

**Keywords:** cellular immunity, humoral immunity, chrysotile asbestos, work experience, professional risk, occupational diseases

## Abstract

The purpose of this research was to study the particularities of the immune status of workers in the field of chrysotile asbestos production, depending on their work experience and professional risk of being affected by chrysotile dust. The research covered 125 men, who were workers at the only enterprise dealing with the extraction and beneficiation of chrysotile ores in Kazakhstan. Indicants of cell immunity were detected by flow cytometry; IgA, IgM, and IgG were detected by a multiplex immunological assay. It was found that, among workers impacted by chrysotile asbestos for more than 15 years, compared with individuals who were not impacted by asbestos dust, the level of CD3+ T-cells was decreased (*t* = −8.76, *p* < 0.001), as well as the number of CD4+ T-cells (U = 1246.0, *p* < 0.001). Moreover, CD8+ T-cells increased (*t* = 5.308, *p* = 0.001), and neutrophil phagocytic activity also increased, by 1.2 times (U = 305.5, *p* < 0.001). It was found that working under the condition of professional contact with chrysotile asbestos dust modifies the indicants of humoral immunity, IgA, IgM, and IgG, to a lesser extent than those of cellular immunity.

## 1. Introduction

Among the problems faced by health authorities in the sphere of health protection and improvement for employees of industrial enterprises, one of the leading positions is occupied by a number of issues in the early detection of occupational diseases, health improvement among workers, and the improvement of labor conditions, which might benefit a significant number of employees in the industrial sphere [1,2,3].

In particular, diseases caused by the impact of industrial aerosols, causing significant economic harm, represent a major concern in the field of occupational diseases around the world, including the Republic of Kazakhstan [4,5,6,7,8].

Pulmonary dust diseases have, as a rule, a chronic form, which is conditioned by the fact that in the primary stages they are asymptomatic, both clinically and radiologically, and also by the fact that functional and immunological tests used in periodic medical examinations are insufficient for the early diagnosis of dust bronchitis and fibrosis caused by different types of industrial fibrogenic aerosols [9,10,11].

Currently, great importance is given to immune mechanisms and cytokine regulation in the development of the proliferative stage of inflammation, in the process of bronchial remodeling in diseases of the lungs of dust etiology [12,13].

As is known, dust impact, including that of chrysotile asbestos, leads to the mobilization of alveolar macrophages, accompanied by the activation of free radical oxidation and the release of mediators, stimulating fibroblast proliferation and collagen synthesis [14,15,16]. Exposure to toxic chrysotile asbestos fibers causes particular stress for macrophages and can lead to the involvement of neutrophilic granulocytes, which is a critical step that leads to an adverse inflammatory response [17,18].

Considering the important role of immunological mechanisms in the development of occupational lung diseases from exposure to fibrogenic dust, associated with the phagocytosis of dust particles and accompanied by the activation of free radical oxidation, we studied the functional state of cell and humoral immunity, with a parallel assessment of the neutrophil phagocytic activity (NPA) of peripheral blood. Thus, this work allowed us to study the features of the immune status in workers in the field of chrysotile asbestos production, depending on the length of work, for the subsequent early detection of professional risk.

## 2. Materials and Methods

### 2.1. Examined Population and Ethics Disclaimer

The research covered 125 men, who were workers at the only enterprise dealing with the extraction and beneficiation of chrysotile ores in Kazakhstan. Workers who no longer worked were not included in the study. All the participants were living in Zhitikara town, Kostanay region, Kazakhstan. The period of work of participants varied from 2 to 41 years. The subjects of the study were only healthy workers in chrysotile asbestos production who were not registered at the dispensary as having any health conditions. Before the study, all employees of the company underwent a mandatory annual medical examination.

The company produces chrysotile asbestos ore via the open pit method at the Zhitikarinskiy deposit. Currently, the geometric parameters of the quarry are as follows: length—4 km; width—1.3 km; depth—270 m. The technology of the mining operations is cyclical, with the use of drilling and blasting operations. In total, 215 workers are employed in mining (11 of them are women), and the average duration of work experience is 13.83 years; SD = 10.1; Me = 12.0. After excavation, the ore is transported by railway to the processing complex. Ore crushing, chrysotile asbestos enrichment, production, storage, and the shipment of varietal chrysotile and inert materials, are carried out in the processing complex. The processing complex employs 311 workers (including 131 women), and the average duration of work experience is 13.2 years; SD = 10.8; Me = 11.0. In total, 196 people (46 of them women) are employed in auxiliary units not engaged in the extraction and enrichment of chrysotile asbestos ores, and they do not have potential professional contact with chrysotile works and the average duration of work experience is 12.27 years; SD = 11.3; Me = 8.0.

The company has a shift mode of operation. All employees have a cyclic shift schedule: 11 h day shift, 11 h night shift, 48 h off, repeat.

The company’s number of workers at the time of the study was 1174, of which men represented 852 and women 322. All the employees were of working age, from 20 to 59 years. Workers (categorized as those engaged in labor with an energy consumption of 201 to 250 kcal/h) who experienced contact with harmful factors of production, totaled 722: men—534, women—188. For the selection of participants for the study, exclusion criteria were consistently applied to the group of male workers (*n* = 534):

1. Results of the medical examination (chronic diseases, needs an in-depth investigation, needs a dispensary)—145 workers;
2. Health conditions with loss of earning capacity in the last year—69 workers;
3. Change of profession in the last 2 years—41 workers;
4. Interruptions in work (labor leave, qualification, or other) in the last 6 months—42 workers;
5. Constant tobacco smoking in the last year—103 workers;
6. Ethical refusal—9 workers.

This study was conducted at the Institute of Public Health and Occupational Health of the Karaganda Medical University. Approved by the Bioethics Committee of Karaganda Medical University (Decision No. 2, dated 14.08.2020), all studies were carried out after obtaining written informed consent from each participant.

### 2.2. Distribution of Trial Arms

Participants were subdivided into trial arms 1, 2, and 3 as follows:

- Group 1: 51 employees directly involved in the extraction and beneficiation of chrysotile asbestos ores, with work experience of less than 15 years (average work experience 8.55 years; SD = 4.5; Me = 8.0), at the age of 22 to 58 years old (average age 36.41; SD = 9.0; Me = 35.0);
- Group 2: 39 employees directly involved in the extraction and beneficiation of chrysotile asbestos ores, with work experience of more than 15 years (average work experience 24.54 years; SD = 6.7; Me = 23.0), aged between 39 and 63 years old (average age 52.49; SD = 7.0; Me = 52.0);
- Group 3: control group, 35 employees not involved in the extraction and beneficiation of chrysotile asbestos ores, without possible professional contact with chrysotile (average work experience 12.37; years SD = 7.1; Me = 12.0), aged between 23 and 60 years old (average age 35.94; SD = 8.8; Me = 35.0).

### 2.3. Hematologic Studies

Venous blood was taken from all participants from the cubital vein, in the morning and strictly on an empty stomach, using a vacuum EDTA tube. Storage and transportation were carried out strictly at 18–20 °C in a vertical position for no more than 12 h. An automatic hematology analyzer, Mindray BC-3200 (Shenzhen Mindray Bio-Medical Electronics Co., Ltd., Shenzhen, China), was used to determine the parameters of the general blood test (hemoglobin, leukocytes, erythrocytes, platelets).

Indicants of immune status, T-cells (CD3+, CD4+, CD8+, immunoregulatory index CD4+8+), B-cells (CD19+), natural killer (NK) cells (CD3+56/16+), TNK cells (CD3-56/16+), and neutrophil phagocytic activity (hereinafter, NPA) were defined by the method of flow cytometry with a cytometer, from Cyflow Space (Partec GmbH, Munster, Germany). For levels of IgA, IgM, and IgG, a multiplex immunological assay with XMap technology on a Bioplex 3D (Bio-Rad Laboratories, Inc., Hercules, CA, USA) was used. Monoclonal antibodies from Beckman Coulter (Beckman Coulter Inc., Brea, CA, USA) were used for combined dying, according to the instructions of the manufacturer, with a no-wash procedure of lysis fixation using Optilyse solution (Beckman Coulter Inc, CA, USA).

### 2.4. Statistic Analysis

During descriptive statistical processing for the total sample and for separate groups, the following calculations were performed: the mean values (M), errors of the mean value (m), standard deviation (SD), and median (Me). The normality of the distribution was determined by the Shapiro–Wilk test; the homogeneity of the dispersions was evaluated according to Levene. The analysis of differences between the mean values was carried out using the nonparametric Kruskal–Wallis test, and for groups with differences in variance, the Welch test was performed. For unequal variances, the Welch test more accurately estimates the true distribution of statistics; differences were considered significant if *p* < 0.05. Multiple a posteriori comparisons of indicators in groups in which there were differences in the means were carried out according to the Games–Howell criterion (considered significant if *p* < 0.017); for indicators “CD 4+, %.” and “NPA, %”, it was performed via Mann–Whitney. The Mann–Whitney U-test is independent of outliers, and concentrates on the center of distribution. A posteriori comparisons with the control group were made according to Dunnett’s test (considered significant if *p* < 0.017). Direct standardization of indicators in groups was applied by age and period of work. All the statistical analyses were performed using the statistical software IBM SPSS Statistics v. 28.0 for Windows (IBM Corp., Armonk, NY, USA).

## 3. Results

### 3.1. Results of Cell Immunity Study

The results of studies of immune status and cellular immunity indicants among participants by group are presented in Table 1.

As we see in Table 1, workers with work experience of less than 15 years (group 1) have decreased total numbers of CD3+ T-cells compared with the control (group 3) (*t* = −6.169, *p* = 0.009); the percentage of CD4+ T-cells is significantly decreased (*t* = −7.792, *p* < 0.001; U1-2 = 697.5, *p* = 0.015). Moreover, the quantity of CD8+ T-cells is significantly increased (*t* = 5.551, *p* < 0.001). The number of CD4+8+ cells is decreased among workers of group 1, with a work period of less than 15 years, compared with group 3, which does not reach the declared level of significance (*t* = −0.513, *p* = 0.022). The level of NPA in group 1, compared to the control, is significantly increased by 1.2 times (U = 428.5, *p* < 0.001).

For workers with more than 15 years of professional contact with asbestos (group 2), compared with the control group (group 3), the level of CD3+ T-cells, as in the previous group, is significantly decreased (*t* = −8.76, *p* < 0.001), and the number of CD4+ T-cells is significantly decreased (*t* = −11.675, *p* < 0.001). Moreover, CD8+ T-cells, as in group 1, are increased (*t* = 5.308, *p* = 0.001). The trend of a decrease in index CD4+8+ in group 2, as in group 1, was not statistically significant (*t* = −0.389, *p* = 0.123).

NPA in workers of group 2, with a working period of more than 15 years, as with workers of group 1, working for a period of less than 15 years, compared with the control, is significantly increased, by 1.2 times (U = 305.5, *p* < 0.001).

### 3.2. Results of Study of NK Cells and Humoral Immunity

A comparison of B-cells (CD19+), natural killer (NK) cells (CD3+56/16+), TNK cells (CD3-56/16+), lymphocytes, and indicators of humoral immunity is displayed in Table 2 and Table 3.

Thus, in Table 2, we see that, in workers with a working period of less than 15 years (group 1), together with the suppression of CD3+ T-cells, the level of CD19+ B-cells is not decreased significantly (*t* = −2.589, *p* = 0.045). In group 1, there is a true decrease in natural killer CD3+56/16+ (NK) cells (*t* = −1.27, *p* = 0.011). In group 2, the level of lymphocyte CD19+ B-cells, compared with the control, is significantly decreased (*t* = −4.039, *p* = 0.002), which is slightly larger compared to group 1, and natural killer CD3-56/16+ (TNK) cells are, vice versa, significantly increased (*t* = 5.893, *p* = 0.002).

The number of immunoglobulins of class IgA, IgM, and IgG in the two studied groups was not noticeably changed, compared with group 3; however, for the dynamics of changes in real values of immunoglobulins, one may find the following: the levels of IgA and IgM tended to decrease, and IgG tended to increase.

## 4. Discussion

It is known that the number of CD4+ T-helper cells and CD8+ cytotoxic T-cells is subject to physiological fluctuations, depending on the biorhythm phase or force and quality of loading factors, and that the immunoregulatory index CD4+8+ (ratio of T-helper cells/T- cytotoxic T-cells) is a more stable indicant, less impacted by physiological data and characterized by higher sensitivity. A decrease in the CD4+8+ index might be observed both at different stages of the normal inflammatory process and with the increased functioning of the immune system, when the amount of CD4+ T-cells in the blood is decreased and CD8+ T-cells are increased, which is observed in the workers of group 1 (CD4+8+ = 1,107, SD = 0.852, Me = 1.1).

Thus, considering the decrease in the immunoregulatory index against the background of increased NPA, we might suggest an impact on the system of cell immunity, accompanied by a decrease in the regulatory function of CD4+ T-cells, and an increase in CD8+ T-cells in terms of the chronic influence of chrysotile asbestos dust; this does not exclude the cytotoxic nature of the cell immunity change among people who experience prolonged contact with chrysotile.

Despite the unidirectionality in changes in indicants of cell immunity, both in group 1 and in group 2, we emphasize several differences in the intensity of change in the number of CD4+ T-cells. CD4+ T-cells are more decreased in group 2, compared with group 1 (U = 697.5, *p* = 0.015). As for the change in the level of CD8+ T-cells, differences in the decrease are similar in the two examined groups (GH = 0.243, *p* = 0.984). This is likely due to the more significant increase in CD4+ T-cells and the absence of a progressive increase in CD8+ T-cells in workers of group 1 compared with group 2; CD4+8+ is decreased, but less evidently, with a working period of less than 15 years, compared with a working period of more than 15. Moreover, NPA is increased in the two groups at the same level.

Considering the progressive decrease in T-helpers CD4+, which are the regulating components in the activation of different cytokines, with the background of similar NPA and similar levels of CD8+ T-cells, we might conclude that workers with a working period of more than 15 years obtain a new level of adaptation, which functions for the examined cell immunity in terms of the chronic influence of dust.

In this study, we revealed that humoral immunity and, in particular, levels of immunoglobulins of class IgA, IgM, and IgG are not changed in groups of workers under the occupational impact of chrysotile asbestos.

Changes in the total amount of immunoglobulins and levels of immunoglobulins of different classes and subclasses in the blood are not normally correlated with the presence of B-lymphocytes in the blood, and this is why the indicants of immunoglobulins of different classes in the blood are independent parameters [19].

The trend towards a change in the level of IgG immunoglobulin with work experience both up to 15 years and more than 15 years, is not significant. The possibility of cytotoxic effect of chrysotile asbestos dust is not determined by an increase of phagocytosis in terms of its chronic impact, which is evidenced by the results of NPA. As is known, there is a possibility of the reaction of immune cytolysis with IgG under the development of aseptic inflammation [19,20], and this tends to increase the antibody-dependent cellular cytotoxicity of chrysotile dust. In our previous work [21], it was shown that chronic exposure to chrysotile asbestos dust has a cytotoxic effect, accompanied by the activation of the phagocytic link of the lungs (alveolar macrophages) and membrane-destructive changes in neutrophils in bronchoalveolar lavage in experimental animals. Pneumofibrosis develops, due to the cytotoxic and membrane-damaging effect of chrysotile asbestos dust.

It is known that in extreme situations, when lung macrophages and the system of mononuclear phagocytes in general fall into a state of depression, the effect of chronic inflammation is developed under the influence of viruses, toxic types of dust, and tobacco use (particles of silica, beryllium oxides, cadmium, nickel, etc.) [22]. In this case, IgA is the most important effect factor in the mucosal lymphoid tissues in the system of mucous tissue of the respiratory ducts [23,24], and it tends to decrease, which might also confirm the impact of dust cytotoxicity.

Summarizing the obtained results of the study of the immune status of workers with a working period of more than 15 years, it is possible to conclude that a change in the indicants of specific cell immunity—of the total number of CD3+ T-cells and CD4+ T-cells, with an increase in CD8+ T-cells and decrease in immunoregulating index CD4+8+ against the background of an NPA increase—might be explained from the viewpoint of the cell-mediated cytotoxicity of chrysotile asbestos dust, and might be examined as an early indicant of the unfavorable impact of chrysotile. As is known, immunological techniques are helpful in litigious or difficult cases for determining such an impact of working-condition factors [25]. Levels of immunoglobulins of the IgA, IgM, and IgG classes are subject to a lesser impact of chrysotile asbestos, compared with cell immunity.

### Limitations of the Study

The sample of workers presents some limitations in the study, as our sample was relatively small. We sought to reduce confounding factors to closely match occupational performance and worker health.

The factor of professional exposure to asbestos was established based on the results of the mandatory certification of workplaces, according to working conditions. The certification was carried out for all professions (workplaces) in the enterprise. Employees of “group 1” and “group 2” had documented professional contact with “asbestos-containing dust”, and employees of group 3 were excluded from this professional contact. The degree of exposure was assessed by the number of years worked. A quantitative assessment of the impact of asbestos fibers has not been carried out.

In addition to the occupational risk of exposure to asbestos, workers also have an environmental risk. The source of risk from the environment is the quarry (Zhitikarinskoye deposit of chrysotile asbestos), next to which the city of Zhitikara is located. All 125 workers are residents of this city. Nevertheless, we believe that the occupational exposure to asbestos from mining and enrichment is disproportionately greater than exposure within the environment.

As such, our ability to generalize to other workers is limited. The small sample size in this study precluded adjustment for possible confounders, reducing the certainty. Additional studies with larger samples and an individual quantification of asbestos exposure will be required to confirm the results presented here.

## 5. Conclusions

In apparently healthy workers with a long working period in terms of professional impact from chrysotile asbestos dust, the indicants of the functional impact on the immune system include an increase in neutrophil phagocytic activity and changes in specific cell immunity—a decrease in the total amount of CD3+ T-cells and CD4+ T-cells, an increase in CD8+ T-cells, and a decrease in the immunoregulating index of CD4+8+.

## Figures and Tables

**Table 1:** Comparative analysis of indicators of T-cellular immunity of three groups of workers in chrysotile asbestos production.

Indicators, %; (Ref)	Group 1 (*n* = 51)			Group 2 (*n* = 39)			Group 3 (*n* = 35)			Post Hoc Comparisons	*p*-Value
	M	SD	Me (95% CI)	M	SD	Me (95% CI)	M	SD	Me (95% CI)		
CD3+, % (55.0–84.0)	65.75	7.11	65.00 (63.75; 63.75)	63.15	8.65	63.00 (60.35; 65.96)	71.91	5.81	69.00 (69.95; 74.05)	GH_1–2_ = 2.591 t_1–3_ = −6.169 t_2–3_ = −8.760	0.288 <0.001 * <0.001 *
CD4+, % (30.0–57.0)	34.29	7.01	34.00 (32.32; 36.37)	30.41	6.35	31.00 (28.35; 32.47)	42.09	5.53	43.00 (39.94; 43.70)	U_1–2_ = 697.5 t_1–3_ = −7.792 t_2–3_ = −11.675	0.015 <0.001 * <0.001 *
CD8+, % (16.0–35.0)	31.29	8.18	31.00 (28.99; 33.59)	31.05	5.04	31.00 (29.42; 32.69)	25.74	4.37	26.00 (24.36; 27.40)	GH_1–2_ = 0.243 t_1–3_ = 5.551 t_2–3_ = 5.308	0.984 <0.001 * 0.001 *
CD4+8+, % (0–1.5)	1.107	0.852	1.100 (0.867; 1.347)	1.232	1.213	0.900 (0.839; 1.625)	1.620	0.537	1.600 (1.423; 1.802)	GH_1–2_ = −0.123 t_1–3_ = −0.513 t_2–3_ = −0.388	0.849 0.022 0.123
NPA, % (20.0–60.0)	41.33	7.121	40.00 (39.33; 43.34)	41.64	6.458	40.00 (39.55; 43.73)	35.14	4.710	36.00 (33.74; 36.96)	U_1–2_ = 1056.0 t_1–3_ = 5.980 t_2–3_ = 6.288	0.614 <0.001 * <0.001 *

Note: ref—reference values; (CD3+, CD4+, CD8+)—cell populations of T-lymphocytes; CD4+8+—immunoregulatory index; NPA—neutrophil phagocytic activity; M—mean; SD—standard deviation; Me—median; CI—confidence interval; GH_1–2_—Games–Howell criterion for groups 1 and 2; U_1–2_—Mann–Whitney criterion for groups 1 and 2; t_1–3_—Dunnett’s test for groups 1 and 3; t_2–3_—Dunnett’s test for groups 2 and 3; *—statistically significant; post hoc comparisons: differences at the level of *p* < 0.017.

**Table 2:** Comparative analysis of indicators of B- and NK cells of workers of three groups in chrysotile asbestos production.

Indicators, %; (Ref)	Group 1 (*n* = 51)			Group 2 (*n* = 39)			Group 3 (*n* = 35)			Post Hoc Comparisons	*p*-Value
	M	SD	Me (95% CI)	M	SD	Me (95% CI)	M	SD	Me (95% CI)		
CD19+, % (6.0–24.0)	14.04	5.657	14.00 (12.45; 15;63)	12.59	5.674	12.00 (10.75; 14.43)	16.63	3.687	17.00 (15.36; 17.90)	GH_1–2_ = 1.449 t_1–3_ = −2.589 t_2–3_ = −4.039	0.455 0.045 0.002 *
CD3+56/16+, %; (0.0–5.0)	1.88	1.50	1.50 (1.46; 2.30)	2.50	2.04	1.50 (1.84; 3.16)	3.15	2.72	2.90 (2.22; 4.09)	GH _1–2_ = −0.616 t_1–3_ = −1.271 t_2–3_ = −0.655	0.259 0.011 * 0.292
CD3–56/16+, %; (3.0–15.0)	14.49	5.20	13.80 (13.03; 15.95)	20.23	11.14	18.00 (16.62; 23.84)	14.33	5.596	13.00 (12.41; 16.26)	GH_1–2_ = −5.734 t_1–3_ = 0.158 t_2–3_ = 5.893	0.012 * 0.993 0.002 *

Note: ref—reference values; CD19+—cell populations of B-lymphocytes; CD3+56/16+—populations of natural killer (NK) cells; CD3+56/16+—populations of TNK cells; M—mean; SD—standard deviation; Me—median; CI—confidence interval; GH_1–2_—Games–Howell criterion for groups 1 and 2; t_1–3_—Dunnett’s test for groups 1 and 3; t_2–3_—Dunnett’s test for groups 2 and 3; *—statistically significant; post hoc comparisons: differences at the level of *p* < 0.017.

**Table 3:** Indicators of humoral immunity of workers of three groups in chrysotile asbestos production.

Indicators, %; (Ref)	Group 1 (*n* = 51)			Group 2 (*n* = 39)			Group 3 (*n* = 35)			Kruskal–Wallis Test	*p*-Value
	M	SD	Me (95% CI)	M	SD	Me (95% CI)	M	SD	Me (95% CI)		
IgA, g/L; (0.63–4.84)	2.610	1.215	2.644 (2.268; 2.951)	2.526	1.245	2.912 (2.123; 2.929)	2.866	1.037	3.101 (2.510; 3.222)	1.629	0.443
IgM, g/L; (0.22–2.4)	1.273	0.459	1.360 (1.144; 1.402)	1.294	0.427	1.433 (1.155; 1.432)	1.332	0.421	1.450 (1.187; 1.476)	0.174	0.917
IgG, g/L; (5.4–18.22)	12.66	4.41	12.13 (11.41; 13.90)	13.46	4.03	13.54 (12.15; 14.77)	11.62	3.27	12.69 (10.49; 12.74)	3.035	0.219

Note: ref—reference values; g/L—grams per liter; M—mean; SD—standard deviation; Me—median; CI—confidence interval; post hoc comparisons: differences at the level of *p* < 0.05.

## Data Availability

The data presented in this study are available on request from the first author. The data are not publicly available, due to ethical restrictions.
